# Sunitinib enhances neuronal survival *in vitro* via NF-κB-mediated signaling and expression of cyclooxygenase-2 and inducible nitric oxide synthase

**DOI:** 10.1186/1742-2094-10-93

**Published:** 2013-07-23

**Authors:** Alma Sanchez, Debjani Tripathy, Xiangling Yin, Jinhua Luo, Joseph M Martinez, Paula Grammas

**Affiliations:** 1 Garrison Institute on Aging, Texas Tech University Health Sciences Center, Lubbock, TX, USA; 2 Department of Neurology, Texas Tech University Health Sciences Center, Lubbock, TX, USA

**Keywords:** COX2, NOS2, Sunitinib, NF-κB, Glioblastoma, Tyrosine kinase inhibitor, Neuron, Neuronal injury

## Abstract

**Background:**

Angiogenesis is tightly linked to inflammation and cancer. Regulation of angiogenesis is mediated primarily through activation of receptor tyrosine kinases, thus kinase inhibitors represent a new paradigm in anti-cancer therapy. However, these inhibitors have broad effects on inflammatory processes and multiple cell types. Sunitinib is a multitarget receptor tyrosine kinase inhibitor, which has shown promise for the treatment of glioblastoma, a highly vascularized tumor. However, there is little information as to the direct effects of sunitinib on brain-derived neurons. The objective of this study is to explore the effects of sunitinib on neuronal survival as well as on the expression of inflammatory protein mediators in primary cerebral neuronal cultures.

**Methods:**

Primary cortical neurons were exposed to various doses of sunitinib. The drug-treated cultures were assessed for survival by MTT assay and cell death by lactate dehydrogenase release. The ability of sunitinib to affect NF-κB, COX2 and NOS2 expression was determined by western blot. The NF-κB inhibitors dicoumarol, SN50 and BAY11-7085 were employed to assess the role of NF-κB in sunitinib-mediated effects on neuronal survival as well as COX2 and NOS2 expression.

**Results:**

Treatment of neuronal cultures with sunitinib caused a dose-dependent increase in cell survival and decrease in neuronal cell death. Exposure of neurons to sunitinib also induced an increase in the expression of NF-κB, COX2 and NOS2. Inhibiting NF-κB blunted the increase in cell survival and decrease in cell death evoked by sunitinib. Treatment of cell cultures with both sunitinib and NF-κB inhibitors mitigated the increase in COX2 and NOS2 caused by sunitinib.

**Conclusions:**

Sunitinib increases neuronal survival and this neurotrophic effect is mediated by NF-κB. Also, the inflammatory proteins COX2 and NOS2 are upregulated by sunitinib in an NF-κB-dependent manner. These data are in agreement with a growing literature suggesting beneficial effects for inflammatory mediators such as NF-κB, COX2 and NOS2 in neurons. Further work is needed to fully explore the effects of sunitinib in the brain and its possible use as a treatment for glioblastoma. Finally, sunitinib may be useful for the treatment of a range of central nervous system diseases where neuronal injury is prominent.

## Background

Angiogenesis, the formation of new blood vessels from existing vessels, is tightly linked to chronic inflammation and cancer [1]. A role for angiogenesis in the development of cancer, first proposed by Folkman in 1971, has been extensively documented [2,3]. Regulation of the complex process of angiogenesis is mediated primarily through activation of receptor tyrosine kinases, thus kinase inhibitors represent a new paradigm in anti-cancer therapy [4]. Sunitinib malate (SU11248/Sutent®; Pfizer) is a small molecule receptor tyrosine kinase inhibitor that has potent anti-angiogenic properties [5]. Tyrosine kinase inhibitors, as a class, compete with ATP for binding within the intracellular domain of receptor tyrosine kinases. Multitargeted agents, such as sunitinib, can block a number of tyrosine kinase receptor families including vascular endothelial growth factor (VEGF) receptors (1–3), platelet-derived growth factor (PDGF) receptors, stem-cell growth factor receptor (KIT), fms-related tyrosine kinase 3 (FLT3) and colony stimulating factor 1 receptor (CSF1R) [6]. Sunitinib has shown therapeutic efficacy in advanced renal cell carcinomas and gastro-intestinal tumors and is approved by the Food and Drug Administration for the treatment of these cancers [6]. Because glioblastomas are highly angiogenic tumors, receptor tyrosine kinase-targeted therapy has been the focus of considerable attention as a novel treatment option for patients with this cancer. Recent clinical success with bevacizumab, a humanized monoclonal antibody against the pro-angiogenic protein VEGF, supports the exploration of angiogenesis inhibitors for glioblastoma [7]. Furthermore, preliminary *in vitro* studies showing an apoptotic effect of sunitinib on glioblastoma cells suggest a promising role for this agent in the treatment of this type of brain tumor [8].

Receptor tyrosine kinase inhibitors can, however, exert numerous effects on multiple cell types, affecting immune responsiveness and inflammatory processes. Several reports indicate that these agents have direct effects on inflammatory mediators and processes in the brain and periphery [9-14]. The multi-kinase inhibitor imatinib has immunomodulatory properties and is anti-inflammatory in several mouse models [9,10]. Imatinib has been shown to affect cytokine production by macrophages as well as reducing delayed hypersensitivity in mice [9]. This agent ameliorates neuroinflammation in a rat model of multiple sclerosis by enhancing blood–brain barrier integrity and by modulating the peripheral immune response [14]. Both imatinib and sunitinib can reverse new onset type 1 diabetes in a non-obese diabetic mouse model [12]. Also, the administration of sunitinib reverses immune suppression in tumor-bearing mice and ameliorates vascular inflammation evoked by drug toxicity [15]. Clearly, receptor tyrosine kinase inhibitors have multiple effects on not only vascular cells but also parenchymal cells. To develop sunitinib as a potential treatment for glioblastoma, the effect of this drug on brain-derived neurons requires further study.

Information regarding the direct effects of sunitinib on brain-derived neurons is limited. A study examining the formation of pathologic autophagic vacuoles in the brains of the APP/PS1 double transgenic Alzheimer’s disease (AD) mouse model shows that injection of sunitinib reduces vacuole formation [16]. In that same study, the increase in pathologic vacuole formation evoked in the human neuroblastoma cell line SH-SY5Y by amyloid beta is diminished by sunitinib. On the other hand, sunitinib has been shown to stimulate autophagy in the neuronal-like PC12 cell line, an effect that is mediated by inhibition of the mTOR signaling pathway [17].

Examination of cultured neurons derived from the Tg2576 AD mouse model demonstrates that treatment with SU-5416, a compound closely related to sunitinib, does not affect cell viability but does alter processing of the amyloid precursor protein [18]. To our knowledge, there is no information, to date, as to the effects of sunitinib on primary cultured neurons. The objective of this study is to explore the effects of sunitinib on neuronal survival as well as on the expression of inflammatory protein mediators in primary cerebral neuronal cultures.

## Methods

### Primary cortical cultures and cell treatment

All animal procedures were performed in accordance with NIH “Guide for the Care and Use of Laboratory Animals” and Texas Tech University Health Sciences Center Institutional Animal Care and Use Committee (IACUC) guidelines. Primary neuron cultures were prepared from cerebral cortices isolated from 18-day gestation rat fetuses, as described previously [19] with the following modifications. The cortices were washed three times with Hanks’ balanced salt solution (HBSS), and pipette-triturated in 10 mL Brooks Logan solution. The neuronal cells were plated at a density of 2 × 10^6^ cells per well on six-well poly-L-lysine coated plates using Neurobasal medium containing B-27® supplement (1:50) (catalog number 17504–044; GIBCO/Invitrogen, Carlsbad, CA, USA), antibiotic (100 U/mL penicillin, 100 μg/mL streptomycin), antimycotic (0.25 μg/mL amphotericin B (Fungizone®, catalog number 15290–026; GIBCO/Invitrogen)), glutamine (0.5 mM) and 5-fluoro-2-deoxyuridine (20 μg/mL, catalog number F0503; Sigma-Aldrich, St. Louis, MO, USA), which was added to prevent proliferation of glial cells. On day 5, fresh medium without 5-fluoro-2-deoxyuridine was added. Neuronal cultures were used for experiments after eight to nine days in culture. All cell culture reagents and media were purchased from GIBCO/Invitrogen.

Sunitinib malate (SU-11248) was provided by Pfizer Inc (New York City, NY) and dissolved in phosphate-buffered saline (PBS) to a stock concentration of 2 mM. Reagents were diluted to experimental doses in Neurobasal media. Neurons were treated with sunitinib for 24 h unless indicated otherwise. For experiments involving NF-κB inhibitors, cultures were treated with sunitinib with or without inhibitors for 24 h. Cells were exposed to 25 μM dicoumarol, 5 μM SN50, 5 μM Bay11-7085 or dimethyl sulfoxide (DMSO, 0.25% final concentration). The inhibitors were purchased from Calbiochem (La Jolla, CA).

### Assessment of cell survival and death

Cell survival was determined using the Cell Titer96 AQueous One Cell Proliferation Assay Kit from Promega (Madison, WI). Cells were washed with PBS and incubated with the MTT reagent 3-(4,5-dimethylthiazol-2-yl)-2-5-diphenyl tetrazolium bromide (1:40 dilution) for 5 to 10 min at 37°C. The cells convert the MTT reagent to formazan, which is quantified by colorimetric assay. The formazan product was read at 490 nm. The number of control cells, that is, viable cells not exposed to any treatment, was defined as 100%.

Cell death was assessed using the Cytotoxicity Detection Kit (Roche, Mannheim, Germany). The assay is a colorimetric quantification of cell death based on the measurement of the activity of lactate dehydrogenase (LDH) released into the supernatant of damaged cells *in vitro*. Cell viability in control samples (untreated cells) is defined as 100% and the amount of viable cells in treated samples was expressed as a percentage of the control.

### Assessment of protein levels by western blot analysis

Neuronal cultures were lysed in buffer (20 mM Tris–HCl, pH 7.4, 50 mM NaCl, 0.5% NP-40, 0.5% deoxycholate, 0.5% SDS, 1 mM EDTA, 1 μg/ml aprotinin) containing protease and phosphatase inhibitor cocktail tablets (Roche). Cell lysates were clarified by centrifugation (13,500 *g*) and the supernatant was used for western blot analysis. The total protein concentration was determined using a commercial kit (catalog number 500–0006, Bio-Rad, Hercules, CA, USA) based on the Bradford protein method.

Total protein (20 to 25 μg) was loaded in each lane and separated in 12% SDS-PAGE by electrophoresis. Bands were detected using chemiluminescence with an X-ray film and quantified using Quantity One v4.6 software (Bio-Rad,). Blots were later incubated with stripping buffer at 50°C for 1 h and re-probed with GAPDH or β-actin. Densitometric measurements of bands were normalized to corresponding GAPDH or β-actin levels with control samples set to 1. Treatment values were then expressed relative to control levels. Primary antibodies used include NF-κB p65 (ab7970 1:300), NOS2 (ab15323, 1:300), β-actin (ab6276, 1:1000) from AbCam (Cambridge, MA), COX2 (#160106, 1:300) from Cayman Chemicals (Ann Harbor, MI) and GAPDH (MAB374, 1:1000) from Chemicon (Temecula, CA).

### Statistical analysis

Prism 5.0 software (GraphPad Inc, San Diego, CA) was used for graphical presentation and statistical analysis. Statistical analyses used included Student’s *t*-test and one-way ANOVA followed by *post hoc* multiple comparison tests to compare data among treatment groups. Data are expressed as mean ± standard error of the mean (SEM) of at least three independent experiments. Significance was determined at *P* < 0.05.

## Results

### Sunitinib treatment enhances neuronal survival

**Figure 1:**
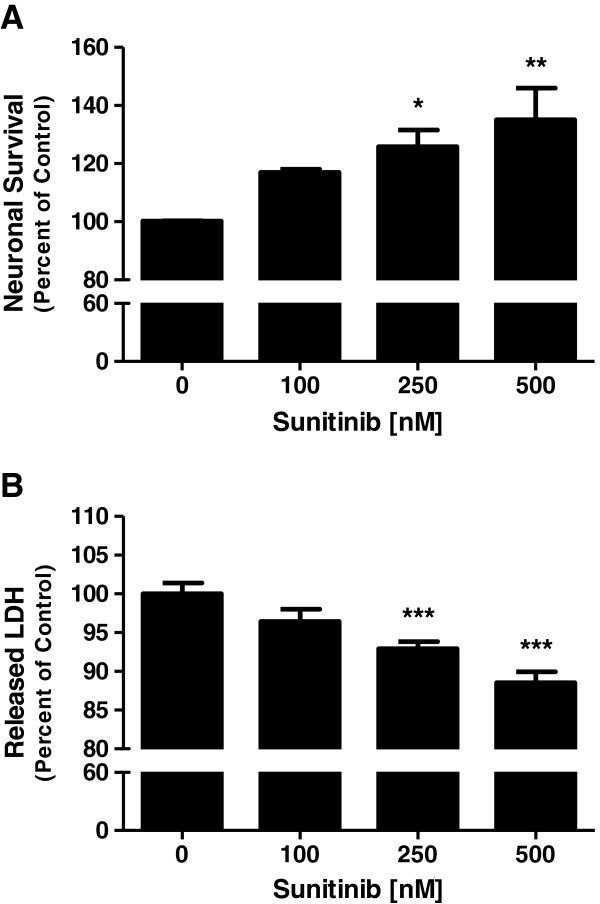
**Survival of neurons treated with sunitinib.** Primary neurons were treated with increasing doses of sunitinib for 24 h. **(A)** Cell survival was measured by MTT assay. **(B)** Cell death was measured by LDH assay. Data are mean ± SEM from six experiments. *P < 0.05, **P < 0.01, *** *P* < 0.001 vs. control. LDH, lactate dehydrogenase; SEM, standard error of the mean.

Neuronal viability was assessed by measuring cell survival as well as cell death. To evaluate survival, primary neurons were incubated with increasing doses of sunitinib (0 to 500 nM) for 24 h and neuronal survival was evaluated by MTT assay. There was a dose-dependent increase in neuronal survival with increasing doses of sunitinib. This increase in cell survival was significant starting at 250 nM (*P* < 0.05) (Figure 1A).

Cell death was determined by measuring the LDH released into the supernatant of sunitinib-treated neuronal cultures. The data showed that LDH release from neurons decreased with increasing sunitinib dose concentration up to 500 nM (*P* < 0.001) compared to untreated neurons cells (Figure 1B).

### Sunitinib increases COX2, NOS2 and NF-κB levels in neurons

**Figure 2:**
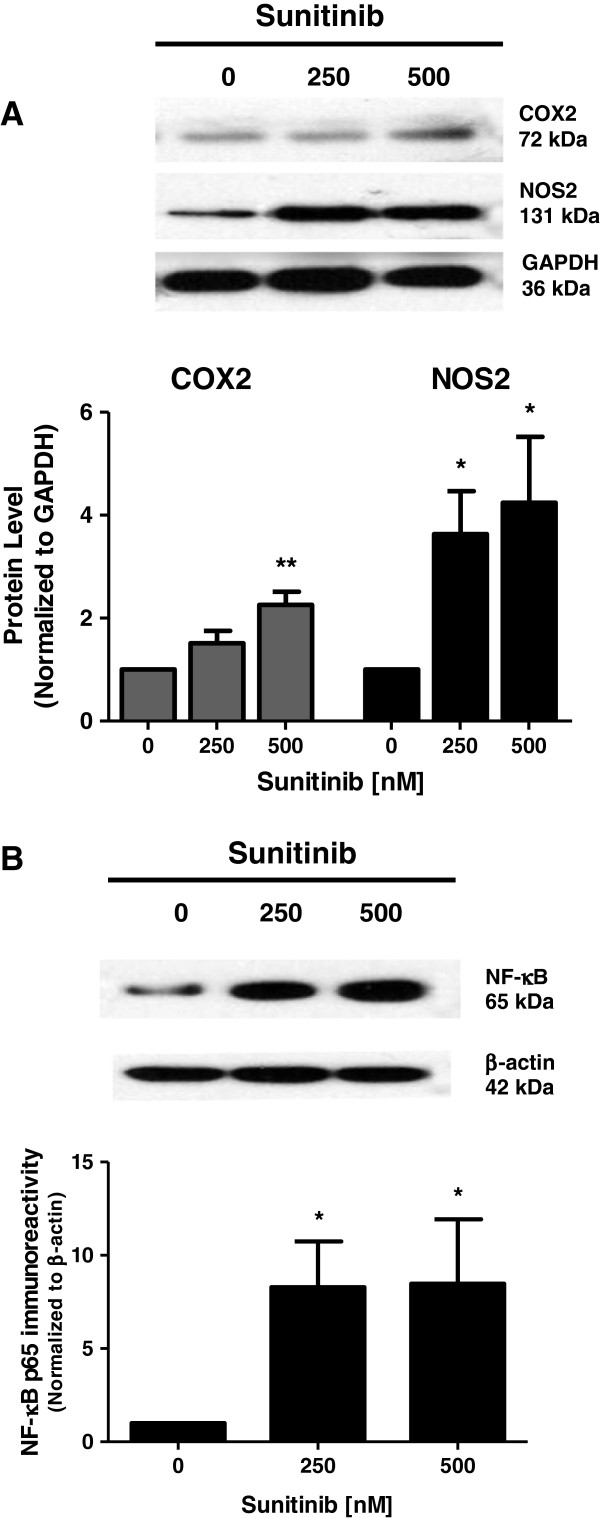
**Expression of COX2, NOS2 and NF-κB in neurons treated with sunitinib.** Neuronal cultures were exposed to increasing dose of sunitinib for 24 h and western blot analysis was used to determine the levels of **(A)** COX2 and NOS2, and **(B)** NF-κB. Data are mean ± SEM from four experiments. *P < 0.05, **P < 0.01 vs. corresponding control. SEM, standard error of the mean.

We determined the ability of sunitinib to affect expression of the inflammatory proteins COX2, NOS2 and NF-κB in primary neuronal cultures. Cortical neurons were exposed to increasing concentrations of sunitinib and expression of these proteins assessed by western blot. There was a dose-dependent increase in both COX2 and NOS2 (Figure 2A). This increase was significant for COX2 at 500 nM sunitinib (*P* < 0.01) and at 250 nM for NOS2 (*P* < 0.05). The expression of NF-κB was determined by examination of the transcription factor subunit p65. Sunitinib caused a significant increase in p65 immunoreactivity at 250 nM (*P* < 0.05). Higher doses of sunitinib did not evoke a further increase in p65 expression (Figure 2B).

### Inhibition of NF-κB signaling attenuates sunitinib-mediated increases in COX2 and NOS2

**Figure 3:**
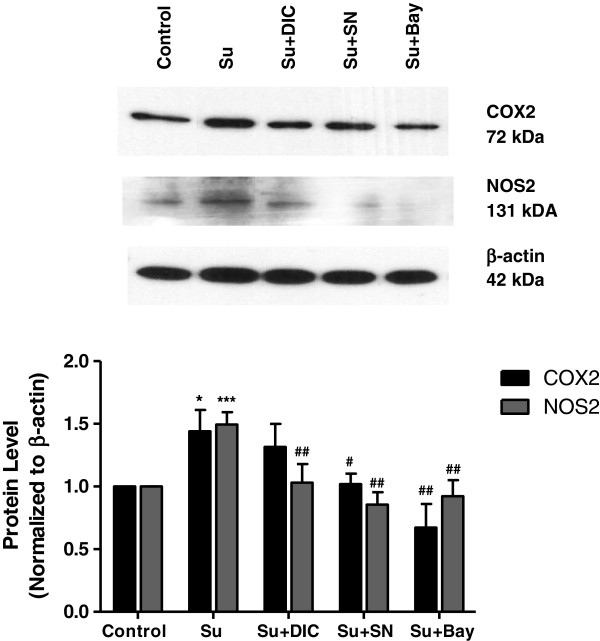
**COX2 and NOS2 expression in neurons treated with sunitinib and NF-κB inhibitors.** Neuronal cultures were treated with sunitinib (500 nM, 24 h) with or without the NF-κB inhibitors dicoumarol (25 μM), SN50 (5 μM) or Bay11-7085 (5 μM). Levels of COX2 and NOS2 were determined by western blot analysis. Data are mean ± SEM from three experiments. *P < 0.05, ***P < 0.001 vs. control; ^#^*P* < 0.05, ^##^0.01 vs. sunitinib alone. Bay, Bay11-7085; DIC, dicoumarol; SEM, standard error of the mean; SN, SN50; Su, sunitinib.

To determine whether sunitinib-evoked increases in COX2 and NOS2 were mediated by NF-κB, three inhibitors of NF-κB activation that target different steps in the activation cascade were employed. SN50 inhibits translocation of active NF-κB to the nucleus while Bay11-7085 inhibits the phosphorylation of IκBα. Dicoumarol alters the cellular redox state. Western blot analysis showed that SN50 (*P* < 0.05 to 0.01) and Bay11-7085 (*P* < 0.01) reduced the sunitinib-mediated increase in both COX2 and NOS2 expression. In contrast, dicoumarol significantly (*P* < 0.01) decreased the sunitinib-mediated increase in NOS2 but did not block the increase in COX2 evoked by sunitinib (Figure 3).

### Inhibition of NF-κB signaling decreases the effect of sunitinib on neuronal survival

The ability of NF-κB inhibitors to affect sunitinib’s neurotrophic effect was assessed by measuring neuronal cell survival (MTT assay) and death (LDH release) in cultures exposed to sunitinib plus NF-κB inhibitors. Primary neurons were treated with sunitinib (500 nM, 24 h) with or without dicoumarol (25 μM), SN50 (5 μM) or Bay11-7085 (5 μM). The specific NF-κB inhibitors SN50 and Bay11-7085 caused a significant reduction in sunitinib-mediated enhancement of cell survival (*P* < 0.01, *P* < 0.001, respectively) (Table 1). Similarly, dicoumarol reduced the increase in cell survival evoked by sunitinib, although the reduction was not statistically significant. Treatment of neuronal cultures with sunitinib plus SN50, Bay11-7085 or dicoumarol resulted in a significant increase (*P* < 0.001, *P* < 0.001, *P* < 0.05, respectively) in neuronal cell death compared to cultures that received sunitinib alone (Table 1).

**Table 1:** **Effect of NF**-**κB inhibitors on neuronal survival and release of lactate dehydrogenase**

	**Neuronal survival**^**a**^	**Lactate dehydrogenase released**^**b**^
Control	100.2 ± 3.8	100.1 ± 3.9
Sunitinib	127.7 ± 7.8 **	85.9 ± 2.9 **
Sunitinib + SN50	98.8 ± 6.1 ^##^	123.9 ± 2.7 ^###^
Sunitinib + Bay11-7085	83.0 ± 7.9 ^###^	123.6 ± 2.7 ^###^
Sunitinib + dicoumarol	110.4 ± 12.6	100.4 ± 5.7 ^#^

^a^Cell survival was measured by MTT assay. Data are mean ± SEM from three experiments.

^b^Cell death was determined by level of released lactate dehydrogenase. Data are mean ± SEM from three experiments.

**P < 0.01 vs control.

^#^*P* < 0.05, ^##^*P* < 0.01, ^###^*P* < 0.001 vs sunitinib alone.

SEM, standard error of the mean.

## Discussion

NF-κB is a key nuclear transcription factor, which is widely expressed and regulates a diverse range of genes involved in cellular inflammation, proliferation and survival [20]. NF-κB is expressed in several cell types in the brain, including neurons, where it regulates neuronal responses to activation by a variety of stimuli [21]. A growing literature documents a complex role for NF-κB-mediated signaling in both physiologic and pathologic conditions. Several studies have shown that activation of NF-κB is associated with degenerative processes in the brain in traumatic brain injuries and in AD [22,23]. Under these pathologic conditions, activation of NF-κB results in overproduction of noxious reactive oxygen species and inflammatory cytokines. Support for the idea that NF-κB mediates deleterious processes in the brain in AD is derived from studies showing that inhibition of NF-κB in AD transgenic mice reduces inflammatory protein expression, improves cognition and affords neuroprotection against the toxic effects of amyloid beta [24,25]. On the other hand, studies have also shown that activation of NF-κB in neurons can promote their survival. There are reports that activation of NF-κB induces expression of genes encoding anti-apoptotic proteins such as Bcl2 and protects neurons against oxidative stresses or ischemia-induced neurodegeneration [25,26]. Exposure of cultured neurons to DNA-damaging compounds causes a reduction in NF-κB, activation of p53 and apoptosis. Pharmacologic interventions that block p53 activation preserve NF-κB expression and protect against apoptosis [27]. Also, inhibition of NF-κB potentiates the neuronal apoptosis induced by amyloid beta [28]. Finally, NF-κB activation appears to be critical to the survival-promoting effects of neurotrophic factors and cytokines [29,30].

In the current study we show that exposure of cultured neurons to sunitinib enhances neuronal survival and that this neurotrophic effect is mediated by activation of NF-κB. In addition, activation of NF-κB results in increases in COX2 and NOS2. These data are in contrast to results obtained with sorafenib, another multitarget kinase inhibitor, which causes a decrease in the expression of NF-κB, NOS2 and COX2 in the brains of AD transgenic mice [25]. However, our study documents the effects of sunitinib in cultured neurons, while the experiments with *in vivo* administration of sorafenib reflect the effect of the drug on the whole brain. It has been suggested that results showing both beneficial and noxious effects subsequent to activation of NF-κB reflect cell-type specific responses [30,31]. In other words, activation of NF-κB in neurons promotes cell survival by inducing pro-survival gene expression while in non-neuronal cells activation of NF-κB causes injury by promoting the release of reactive oxygen species, inflammatory cytokines and excitotoxins.

Both COX2 and NOS2 are closely linked to NF-κB activation and are downstream effectors of this pathway in many cell types. Increased COX2 expression has been observed after NF-κB activation in mixed cortical neuron cultures while in organotypic hippocampal slice cultures, inhibition of NF-κB activity results in decreased COX2 [32,33]. NF-κB has been reported to upregulate the NOS2 gene transcriptionally in hippocampal neurons and in human neuroblastoma cells [34,35]. The multistep activation pathway of NF-κB is subject to regulation at several points. Under resting conditions, NF-κB is sequestered in the cytoplasm by binding to inhibitor protein subunits (IκBs). Activation of NF-κB occurs when IκBα is degraded after phosphorylation and freed NF-κB translocates to the nucleus. The three NF-κB inhibitors used in this study affect different steps in the activation cascade. SN50 inhibits translocation of active NF-κB to the nucleus while Bay11-7085 inhibits the phosphorylation of IκBα [36,37]. Dicoumarol appears to affect NF-κB by a less specific mechanism. Dicoumarol alters the cellular redox state and NF-κB is known to be a redox-sensitive factor [38]. In this regard, the DNA-binding activity of oxidized NF-κB is significantly diminished [39]. Here we document that SN50 and Bay11-7085 inhibit sunitinib-mediated increases in COX2 and NOS2 while dicoumarol inhibits the effect of sunitinib on NOS2 but not on COX2. These results show that both COX2 and NOS2 are downstream of NF-κB activation in neurons but also suggest that regulation of COX2 expression by sunitinib is mediated by additional signaling mechanisms.

As discussed above, in the context of NF-κB, both COX2 and NOS2 have a range of effects in the brain. These proteins have been identified as contributory to neuroinflammatory processes in a number of central nervous system (CNS) diseases. Indeed, a large body of data implicates overexpression of COX2 and NOS2 in the development of cell injury and death in the brain [40-43]. However, despite their role in pathologic neuroinflammation, we found that treatment of neuronal cultures with sunitinib promotes cell survival as well as increased expression of both COX2 and NOS2. These data are in agreement with studies that show beneficial or protective effects of these proteins in neurons. In this regard, a protective role for COX2 has been demonstrated by experiments where adenovirus-mediated COX2 gene delivery confers protection to neurons against DNA damage induced by oxidative, excitotoxic and ischemic stresses [44]. Inhibition of COX has been shown to inhibit induction of long-term potentiation, an index of synaptic plasticity [45]. Also, inhibiting COX2 blocks the increase in brain-derived neurotrophic factor (BDNF) evoked by spatial learning, suggesting that COX2 plays a permissive role in synaptic plasticity and spatial learning via a BDNF-associated mechanism [45]. Prostaglandin D2, a product of COX2, potently rescues hippocampal neurons exposed to glutamate toxicity and organotypic slices from NMDA-mediated injury [46].

There is a large literature implicating elevated nitric oxide, and especially induction of NOS2, in pathologic neuroinflammation and neurodegenerative diseases of the brain [47,48]. However, nitric oxide has also been documented to exert positive (neurotrophic) effects on neurons and appears to play multiple roles in neuroprotection, neurodegeneration and brain plasticity [49-51]. Nitric oxide acting together with BDNF maintains the process of neural differentiation in neural stem cells [52]. Activation of soluble guanylate cyclase by nitric oxide mediates depolarization-induced protection of dopaminergic neurons from the cytotoxicity induced by MPP^+^[53]. In the latter study, treatment of cultures with MPP^+^ decreases the number of dopaminergic neurons whereas cell loss is inhibited by elevated extracellular K^+^, a neuroprotective effect that is attenuated by the nitric oxide inhibitor L-NAME. A beneficial effect of NOS2 on neuronal health is inferred by data showing that deletion of the NOS2 gene in AD transgenic mice is associated with neuronal loss [54]. Nitric oxide has been shown to affect amyloid beta metabolism in a way that promotes degradation of this toxic protein. Specifically, nitric oxide increases the MMP-9/TIMP-1 ratio leading to enhanced degradation of amyloid beta *in vitro*[55]. The physiologic relevance of this *in vitro* observation is supported by data that AD mice crossed with NOS2 knockout animals show decreased MMP activity and increased amyloid burden [55]. Whether nitric oxide is beneficial or noxious in neurons may be determined by length and/or level of exposure. In a neuronal cell line, BDNF and NOS2 expression both increased 4 h after exposure to sodium L-lactate, whereas at 24 h exposure, expression of these proteins diverged with NOS2 increasing but not BDNF [56]. Based on observations that low levels of nitric oxide are neuromodulatory but high levels are neurotoxic, a dual role for nitric oxide, the main product of NOS2, in neuronal survival has been proposed [57].

Treatment for glioblastoma, a highly vascular tumor, has traditionally consisted of radiotherapy and temozolomide-based chemotherapy. However, virtually all patients recur, leading to a fatal outcome. Tyrosine kinase targeted therapy has been the focus of attention as a treatment option for these patients based on the idea that a multifaceted approach that targets both the primary cancer and the vascular angiogenic component might provide additional clinical benefit [7]. In addition, recent data suggest that the multitarget tyrosine kinase inhibitor sunitinib exerts some direct apoptotic effects on glioblastoma cells [8]. However, administration of drugs into the brain is complicated by the need to traverse the blood–brain barrier as well as the number of different cell types in the CNS. Although the ability of sunitinib to cross the blood–brain barrier has been documented [58], information as to the effects of sunitinib on neurons and other cell types in the brain is limited. In the current study, we found that sunitinib increases neuronal survival and that this neurotrophic effect is mediated by NF-κB. It is also worthwhile to note that the dose range of sunitinib that is anti-angiogenic, and as indicated in the current study neuroprotective (nanomolar), is considerably less than the dose shown to be toxic to glioblastoma cells [59,60]. In addition, the inflammatory proteins COX2 and NOS2 are upregulated by sunitinib in an NF-κB-dependent manner. These data are in agreement with a growing literature suggesting beneficial effects for inflammatory mediators such as NF-κB, COX2 and NOS2 in neurons. Further work is needed to fully explore the effects of sunitinib in the brain and its possible use in the treatment of glioblastoma. Finally, sunitinib, as well as other multitargeted receptor tyrosine kinase inhibitors, may be useful for the treatment of CNS diseases where neuronal injury is prominent.

## Conclusions

The current study contributes to the emerging literature that documents neuroprotective effects for NF-κB, COX2 and NOS2, inflammatory proteins that have traditionally been viewed as only deleterious in the brain. This newly described neuroprotective effect of sunitinib suggests an intersection between angiogenic and neurodegenerative processes in the brain. By dissecting the specific cellular responses to sunitinib, and other receptor tyrosine kinase inhibitors, strategies could be developed that enhance the beneficial effects and minimize the deleterious effects of inflammatory proteins in the brain in a variety of pathological conditions.

## Abbreviations

AD: Alzheimer’s disease; BDNF: brain-derived neurotrophic factor; CNS: central nervous system; HBSS: Hanks’ balanced salt solution; LDH: lactate dehydrogenase; PBS: phosphate-buffered saline; SEM: standard error of the mean; VEGF: vascular endothelial growth factor.

## Competing interests

All authors have contributed to the work and agree with the presented findings. This work has not been published before nor is it being considered for publication by another journal. There is no conflict of interest with any of the authors.

## Authors’ contributions

AS performed the cortical culture isolation, carried out the neuronal survival and biochemical assays, ran statistical analysis on the gathered data, drafted the manuscript and participated in the revision of the manuscript for intellectual content as well as the interpretation of data. XY, DT and JL aided AS in cell culture and the execution of laboratory experiments. JMM aided in the drafting of the manuscript and participated in the revision of the manuscript for intellectual content as well as the interpretation of data. PG is the laboratory’s primary investigator, and participated in the design and coordination of the study, aided in the drafting and revision of the intellectual content of the manuscript, interpreted data and gave final approval of the manuscript. All authors have read and approve the final manuscript.
